# An Optical Frequency Comb Tied to GPS for Laser Frequency/Wavelength Calibration

**DOI:** 10.6028/jres.115.028

**Published:** 2010-12-01

**Authors:** Jack A. Stone, Patrick Egan

**Affiliations:** National Institute of Standards and Technology, Gaithersburg, MD 20899-8211

**Keywords:** global positioning system, GPSDO, laser frequency calibration, laser wavelength calibration, optical frequency comb

## Abstract

Optical frequency combs can be employed over a broad spectral range to calibrate laser frequency or vacuum wavelength. This article describes procedures and techniques utilized in the Precision Engineering Division of NIST (National Institute of Standards and Technology) for comb-based calibration of laser wavelength, including a discussion of ancillary measurements such as determining the mode order. The underlying purpose of these calibrations is to provide traceable standards in support of length measurement. The relative uncertainty needed to fulfill this goal is typically 10^−8^ and never below 10^−12^, very modest requirements compared to the capabilities of comb-based frequency metrology. In this accuracy range the Global Positioning System (GPS) serves as an excellent frequency reference that can provide the traceable underpinning of the measurement. This article describes techniques that can be used to completely characterize measurement errors in a GPS-based comb system and thus achieve full confidence in measurement results.

## 1. Introduction

Optical frequency combs are used routinely to generate optical radiation with a frequency uncertainty limited only by the uncertainty of the master clock that sets the repetition frequency of the comb. The fundamental limitation is the uncertainty of the primary cesium clock, currently about 3 × 10^−16^ [1]. Combs are also quite useful in applications that may not require such high accuracy but benefit from the fact that a comb can generate radiation over a broad spectrum of optical wavelengths. In the Precision Engineering Division (PED) of NIST,^1^ we have a need to calibrate laser frequency for lasers with wavelengths ranging from 530 nm to 1.5 μm, by comparison of the laser frequencies to the known frequencies generated by a comb.

Calibration of laser frequency provides equivalent knowledge of the laser vacuum wavelength; we will interchangeably use the terms “frequency calibration” and “wavelength calibration.” Since 2008, all NIST laser wavelength calibrations are traceable (either directly or via a transfer standard) to the comb, and calibrated lasers operating in interferometers form the backbone of modern length metrology. Thus, combbased vacuum wavelength measurements effectively form the top of the traceability chain for most length measurements in the United States.

For purposes of laser calibration, a relative uncertainty of a few parts in 10^12^ is more than sufficient for any of our needs. (The relative uncertainty is the same whether discussing frequency or wavelength.) The most demanding requirement we face is a need to calibrate iodine stabilized lasers operating at 633 nm. Under favorable circumstances, these lasers can reproduce their frequency/wavelength at a level of ≈4 × 10^−12^ [2]. There is no benefit in achieving a calibration accuracy much better than the reproducibility. Other calibrations of interest to us include commercial lasers operating at 543 nm and at 1.52 μm; for these lasers the wavelength reproducibility is much worse, and there is no motivation to achieve a relative uncertainty better than 10^−9^.

A comb referenced to a GPS signal is entirely sufficient to meet these needs. The GPS signal furthermore provides a very clear traceability path to the unit of time as realized by NIST. In effect, the GPS signal, in combination with a comb, is capable of providing the unit of length (vacuum wavelength) delivered by satellite to any point in the world, over a broad range of wavelengths. As combs become more robust and less costly, this “length by satellite” is coming into wide use by National Metrology Institutes (NMIs) and may also become an attractive tool for use in high-level secondary laboratories. Arguably, the traceability path for these GPS-based measurements is particularly straightforward and easy to demonstrate, and a well-functioning comb is capable of achieving uncertainties many orders of magnitude better than what is required for laser calibration. Nevertheless, it is necessary to verify the reliability of the overall measurement process. This paper will describe a suite of tests that we use for the PED comb to assure that the comb indeed delivers order-of 10^−12^ accuracy as required for our calibration needs. Some further discussions of this topic may be found in Ref. [3]. We would argue that this suite of tests—or similar tests–could be used by anyone to achieve essentially complete confidence in their measurement results for uncertainties as small as a few parts in 10^12^. The procedures as described in this article are directed toward users whose uncertainty requirements are not more stringent than this level.

## 2. Implementation of a Comb-Based System for Calibrating Laser Frequency

**Fig. 1:**
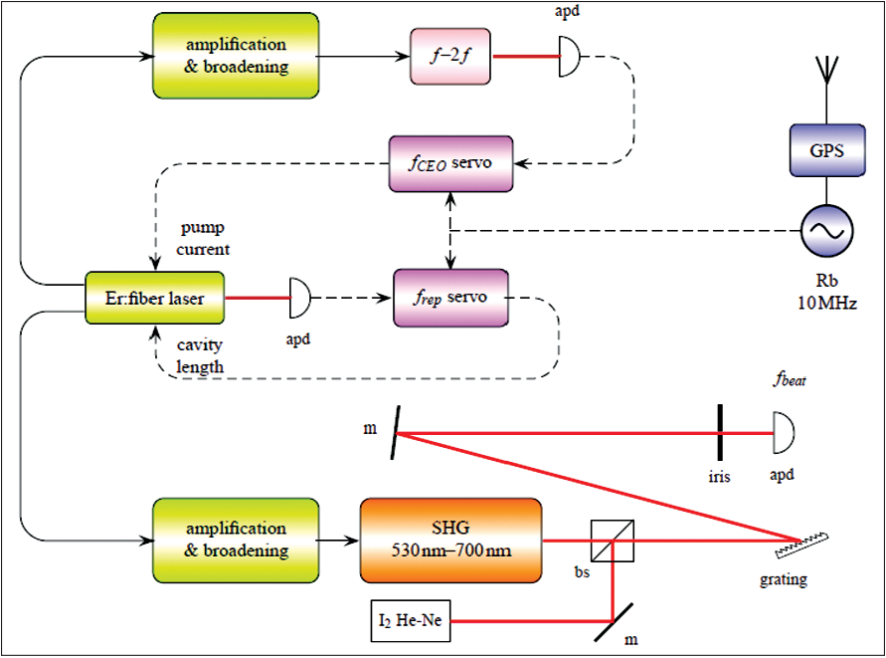
Basic configuration of comb and optics for calibration of I_2_ stabilized lasers. Components shown in the diagram are: **apd**—avalanche photodetector; **SHG**—second harmonic generator; **bs**—beamsplitter; **m**—mirror. Addition optical components not shown in the diagram include waveplates, focusing lenses, and optical isolators.

The operating principles of combs have been described extensively in the literature and will not be discussed in detail here. Several references [4, 5, 6] give good reviews of frequency combs. Fox et al. [7] explicitly discuss using a GPS in conjunction with a comb for laser calibrations, and some aspects of the PED system has been discussed previously by Stone et al. [3]. In this paper we cannot review all aspects of comb operation, but in this section we will provide some needed background information and will describe those elements of our measurement system that may be somewhat different from standard configurations. Figure 1 shows a simplified schematic of the basic components of the frequency comb and of the set-up for calibration of an iodine stabilized laser.

### 2.1 Basis of the Measurement

An optical frequency comb is based on a mode locked laser emitting a uniform train of short pulses with some repetition frequency *f*_rep_. In terms of frequency, the pulse train consists of a set of uniformly spaced frequency components, with frequencies *f_N_* given by

(1) $$f_{N}=f_{\mathrm{CEO}}+N\;f_{\text{rep}},$$

where *f_CEO_* is the carrier-envelope offset frequency, measured in a manner described later. *f_CEO_* can be either positive or negative. *N* is the mode order, a large integer typically greater than 10^6^. When we calibrate the frequency of a laser, the output beam of the comb is combined (via a beamsplitter) with the beam from the laser under test. The power of the combined beam is measured by a fast photodetector, and the output of the detector then contains beat frequencies *f*_beat_ corresponding to the difference in frequency between comb components and the unknown frequency *f*_test_ of the laser under test. Thus, if a beat with one known component *N* of the comb output can be isolated, the frequency *f*_test_ is determined from a measurement of *f*_beat_:

(2) $$f_{\text{test}}=f_{\mathrm{CEO}}+N\;f_{\text{rep}}\pm f_{\text{beat}}.$$

**Fig. 2:**
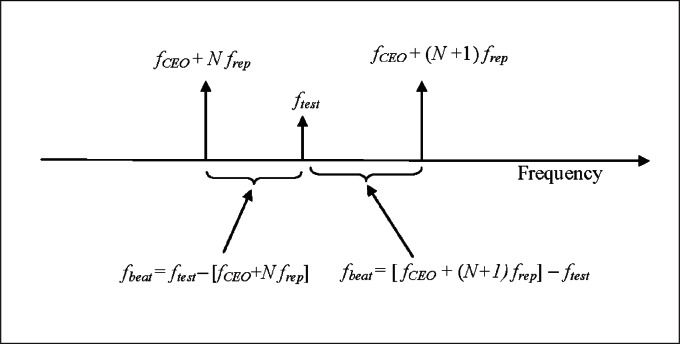
Beat frequencies arising from interference between the test laser and two adjacent comb components. In addition to the two beat frequencies indicated in the diagram, there will be an additional RF signal at frequency *f*_rep_ due to interference between the two adjacent comb components, and there will be many higher frequencies due to interference with additional comb components not shown in the diagram.

In the above equation, *f*_beat_ represents a positive quantity—the measured beat frequency—which must be added or subtracted depending on whether *f*_test_ is larger or smaller than the frequency of the *N*th comb component. The situation is as shown in Fig. 2, where the beat between *f*_test_ and the next-lowest comb component corresponds to a positive sign in Eq. (2), and the beat with the next-highest comb component corresponds to a negative sign.

Signs may be determined by making small changes in the frequencies *f*_rep_ and *f_CEO_* and observing the corresponding change in *f*_beat_. If *f*_beat_ is observed to increase when *f*_rep_ is increased very slightly, then *f*_beat_ must be the beat with component *N* + 1 in Fig. 2, and the negative sign must be used in Eq. (2). Conversely, if *f*_beat_ decreases then the positive sign must be used. Similarly, if *f_CEO_* is increased slightly, and if this has the same effect (in terms of increasing or decreasing *f*_beat_) as does an increase in *f*_rep_, then *f_CEO_* is positive. In a setup such as ours, where both the offset frequency and beat frequency can be servo controlled, the signs are determined by the polarity settings of the servo loops; thus it is not necessary to re-determine the signs for every measurement.

For our system, *f*_rep_ is normally 100 MHz, and we only measure beat signals between 0 MHz and 100 MHz. As shown in Fig. 2, there are always two such signals, and the two frequencies sum to 100 MHz.

The accuracy of measurements rests primarily on achieving a low uncertainty in *f*_rep_. To achieve an uncertainty in *f*_test_ of 1 kHz (≈2 parts in 10^12^), *f*_rep_ must be known with a relative uncertainty of 2 parts in 10^12^, whereas the offset frequency *f_CEO_* (typically 20 MHz, or 40 MHz after frequency doubling to the visible) and the beat frequency *f*_beat_ (less than 100 MHz) need be measured with a relative uncertainty of only 10^−5^.

### 2.2 The Frequency Comb

We use a commercial optical frequency comb [8, 9]^2^ as the basis of our system, with two modifications to the commercial comb. The comb system consists of a mode-locked erbium fiber laser, an *f*-2*f* interferometer for determining offset frequency, and frequency doubling optics to reach visible wavelengths. The erbium laser actually has two outputs that are independently amplified and shaped, and are broadened in two highly nonlinear fibers. (See Fig. 1.) One output is broadened so that is covers a wavelength range of one octave, between roughly 1 μm and 2 μm, and is used for determining the offset frequency *f_CEO_*. Initially, the offset frequency was measured using a two-arm interferometer; frequency-doubled light from a BBO crystal in one arm of the interferometer (with offset frequency 2 × *f_CEO_*) interferes with un-doubled light in the second arm (with offset frequency *f_CEO_*), generating a beat signal at *f_CEO_* for detecting and controlling the offset frequency. (See Appendix A for definitions of acronyms such as BBO.) At present we generate the *f_CEO_* signal in the single arm of the interferometer containing the BBO crystal; light emerging from the crystal contains both doubled and un-doubled components which interfere with each other to create the signal. Although walk-off in the BBO crystal reduces signal strength relative to what might be obtained with PPLN [10], we nevertheless achieve S/N ratios using this method that are similar to what was achieved with the two-arm interferometer (roughly 30 dB to 35 dB as measured with 300 kHz RBW). The single-arm arrangement is advantageous because it is more robust in terms of alignment than is a two-arm interferometer. A feedback loop controlling the pump laser current is used to stabilize *f_CEO_* to a 20 MHz reference frequency that is derived from the GPS signal.

A second output of the erbium laser is independently amplified and broadened in a separate nonlinear fiber. This output can be used directly for calibrations in the IR, or it can be doubled in a PPLN crystal to provide a visible output. Three PPLN crystals suffice to cover a spectral range from 530 nm to 700 nm.

Another modification of our comb system is a mechanism for sliding a glass plate into and out of the freespace region of the comb laser, so as to provide a means for rapidly changing the optical length of the resonator and hence the repetition frequency. This operation is needed to determine *N*, as described later. Although the commercial laser system includes a mechanism based on a piezo-drive motor for making coarse adjustments to the length of the resonator, suitably large shifts in repetition frequency would require an inordinate amount of time. We can rapidly shift the frequency by 208.22 kHz by inserting a 12.7 mm thick piece of BK7 into the free space region of the erbium laser resonator. Losses due to reflections from uncoated surfaces of the glass appear to have little effect on laser operation or on mode locking. When the 12.7 mm piece of glass is inserted into the beam, mode lock is usually re-established immediately and can always be initiated by slightly jiggling the glass.

In passing we note that the change in comb repetition frequency when inserting the glass tells us the change in optical length of the laser cavity, arising from the group refractive index of the glass. If the group index could be determined with low uncertainty, then the absolute thickness of the glass could be obtained from the measured frequency change. The precision of the measurement can be very high (sub-nanometer), limited only by the short-term stability of the free-running comb, and thus this phenomenon could serve as the basis of a technique for measuring sub-nanometer thickness variations of transparent materials.

### 2.3 Reference Frequency (GPSDO)

The repetition frequency (*f*_rep_) of the mode-locked laser is normally phase locked to a multiple of the frequency of a GPS disciplined rubidium oscillator (GPSDO) [11]. Short-term stability is provided by the rubidium oscillator. In a GPSDO, the absolute longterm accuracy is achieved by a servo that adjusts the output frequency to agree with GPS signals. The fundamental accuracy of the system thus rests in the GPS signal, which is traceable to NIST; it is “calibrated” daily in the sense that NIST continuously monitors the signal and publishes performance data on the web [12]. Some attention should be given to placement of the GPS antenna in a position where it views as many satellites as possible and where it is not subject to reflections from nearby buildings. Also, the GPSDO unit should be chosen with some care, as certain units will provide better short-term stability than others. Short-term performance depends both on hardware (particularly the local oscillator) and on the software that must handle switching between satellite signals, weigh the signals appropriately, and implement a steering algorithm to correct the local oscillator output. These issues have been discussed by Lombardi [13, 14, 15].

### 2.4 Controlling the Comb Repetition Rate

A photodiode detects comb pulses at the repetition frequency *f*_rep_, and a fast piezo actuator in the erbium laser is used to control the laser cavity length and thus set the repetition frequency. Normally, the 10th harmonic of the repetition frequency is phase locked to a 1 GHZ reference derived from the GPSDO signal, thus setting the repetition rate to 100 MHz. Under some circumstances—particularly for determining the mode order *N*—it is useful to operate at other repetition frequencies (never far from 100 MHz). This can be done by replacing the GPSDO signal with a variable reference frequency provided by the output of a synthesizer/signal generator. To preserve accuracy, the time-base of the synthesizer/signal generator must be provided by the GPSDO signal. Good performance will not be obtained unless the signal generator has very low phase noise. When the comb repetition frequency is thus controlled, measurements of the highest accuracy can be obtained in a convenient manner, and we use this mode of operation for many of our measurements.

### 2.5 Controlling the Beat Frequency

We also use a second mode of operation where *f*_rep_ is not held fixed but is servoed so as to keep the beat frequency *f*_beat_ constant. This alternate approach provides some operational advantages over a signal generator (and is much less expensive to implement). The primary advantage of this method is that it can be implemented in a scheme that will automatically establish servo lock with little or no operator intervention, which is important when it is necessary to rapidly and repeatedly change the comb repetition frequency. We use this approach when measuring the mode order as described in Sec. 2.7. Keeping the beat frequency constant also makes it possible to improve signal to noise with the aid of a narrow filter, as described in Sec. 2.6. A frequency lock that is easy to implement and has a wide capture range is described in Ref. [16]. We have tried several variations on this scheme.

There are several possibilities for straightforward implementation of a frequency lock with a unique lock point, which will provide automatic lock acquisition with minimal requirements for operator intervention. An electrical high pass or low pass filter with cutoff near the desired beat frequency can be used as a discriminant for measuring and controlling *f*_beat_. The power transmitted through the filter varies rapidly near the cutoff frequency. Satisfactory frequency control can be achieved simply by servoing the comb repetition frequency so as to hold the power of the filtered signal constant at some threshold value; that is, the PI controller that normally holds *f*_rep_ constant is instead used to hold the filtered power equal to the threshold value. The control point will change slightly if laser power varies, but the effect is not severe if the filter rolloff is sufficiently sharp.

A system with reasonable performance can be built simply by plugging together inexpensive connectorized stock components. The signal is first pre-filtered to select the range between 50 MHz and 100 MHz, so that only one beat signal is present. Two stock 70 MHz low-pass filters can be cascaded to provide a sharp discriminant, suitable for stabilizing the beat frequency at the center of a stock 70 MHz bandpass filter. (This will simultaneously stabilize the signal at *f*_rep_–*f*_beat_ to the center of a 30 MHz bandpass filter.) A splitter, mixer, and low pass filter can be used to generate a DC signal proportional to the power transmitted through the filter. If the beat signal has constant amplitude, we see variations in the lock point over the course of an afternoon of less than ±200 kHz, and the 1-second Allan deviation is 6 kHz. A 10 % change in the amplitude of the beat signal shifts the lock point by about 400 kHz.

An order of magnitude better performance can be achieved using a power-insensitive scheme that depends on the phase shift of the filtered signal, but this method does not result in a unique lock point. As described in Ref. [16], comparing the filtered power to the total unfiltered power will give a lock point insensitive to power fluctuations, but a unique lock point will not be achieved without careful attention as to how signal levels behave at the edges of the pre-filter passband. For our application, the potentially tighter lock provided by power-insensitive schemes is of no clear benefit, whereas it is of primary importance to achieve reliable automatic locking, requiring a unique lock point.

The main drawback of stabilizing the beat frequency, rather than the repetition rate, is that this approach requires very careful measurement of the *repetition* frequency, which is no longer controlled by the repetition rate servo. This will require long averaging times and will severely test the performance of a frequency counter. Actually, the counter *does not* need an extremely accurate internal timebase, because achieving suitable accuracy of the timebase is *only* possible via using the GPSDO as an external timebase for the counter. But good timing resolution is required in order to achieve accurate measurements in short periods of time. For example, with 1 ns timing resolution, a 1 s frequency measurement has a resolution of only 1 part in 10^9^, which may not be sufficient for purposes of determining mode order. Thus, it is necessary to purchase a counter that has good timing resolution and it is necessary to use long measurement samples to achieve high accuracy. This point is discussed further in Sec. 4.2. In any event, if measurements with the beat frequency locked agree well with results obtained with the repetition frequency locked, this can serve as a cross check to give confidence in both the frequency counter and in the *f*_rep_ servo.

### 2.6 Beat Frequency Measurement

To measure the beat frequency, the laser beams from the comb and test laser are combined at a beamsplitter and the time-varying power in the superimposed beams (at frequency *f*_beat_) is detected with an avalanche photodetector. The primary difficulties with measuring *f*_beat_ arises from the fact that only a very small fraction of the comb power is in the mode that gives rise to a beat signal. The problem is particularly a concern for us because of the relatively low repetition frequency of our comb laser (100 MHz) and the corresponding high density of comb lines. After frequency doubling to the red, the output power of the comb is a few milliwatts spread over several nanometers spectral width. This spread corresponds to 1.5 × 10^4^ comb lines, only one of which will contribute to the signal *f*_beat_ that we wish to measure. The rest of the comb power degrades the signal through its contribution to shot noise and through saturation of the detector. Depending on the detector and its built-in amplification, saturation may occur due to either the DC comb power or due to RF signals at *f*_rep_ (and multiples of *f*_rep_) arising from interference between adjacent comb lines, because these signals are much larger than is the signal at *f*_beat_. If too much amplification is built into the detector, it will be difficult to avoid saturation. Saturation can be avoided if the detector has lower amplification and its output is filtered prior to additional stages of amplification.

Several strategies are needed to maximize signal to noise and reduce detector saturation effects. Probably the most important single step is simply to take great care in aligning the beam from the test laser with the beam from the comb. To be more precise, what is ideally needed is perfect matching of the wavefronts–two coaxial Gaussian beams with the same waist position and same size, traveling in the same direction. The ultimate method for assuring matching of the wavefronts—and rejecting unmatched portions of the beams that do not contribute to signal but contribute to saturation and noise—is to couple both beams into a single mode fiber prior to the detector. If a fiber is not used, it must be assured that the beams overlap very well, travel accurately in the same direction, and have similar waist size (or equivalently, similar spot sizes in the far-field region). A factor of two mismatch in the waist size will result in a 1.9 dB reduction in signal strength (calculated from the overlap integral assuming that the beams are Gaussian and both can be focused completely onto the detector). For beams of equal size, there will also be >1.9 dB reduction in signal if the beams are misaligned by an angle greater than 2/3 of the beam divergence (half angle), or if one beam is offset laterally from the other by more than 2/3 of the waist radius, or if the positions of the waists are separated along the direction of propagation by a distance greater than the Rayleigh range.

Other things can be done that will slightly improve signal to noise and will reduce the potential problem of detector saturation by the comb power. First, a narrow-band optical filter or grating can be used to reject unwanted spectral components of the comb signal. A grating in Littrow configuration provides potentially better performance than do optical filters and, unlike the narrow band filter, works over a range of wavelengths. The grating alleviates the problem of detector saturation and slightly improves signal to noise.

Similarly, a non-symmetric beamsplitter or polarizing optics can be used to combine the comb and test laser beams in proportions other than 50 %, which is slightly advantageous when the test laser has much less power than the comb (an iodine stabilized laser, for example). If the test laser has 1/10 of the power of the comb prior to mixing, then S/N is optimized (assuming shot noise) by a splitting ratio of 75 % to 25 % in favor of the test laser. The improvement in S/N relative to a 50 % beamsplitter is almost negligible—only 1 dB— but the total power on the detector is reduced by 40 % and thus helps avoid saturation.

Perhaps the simplest and most effective way of improving S/N is to limit the detection bandwidth for the electronic signal going to the frequency counter. We typically stabilize *f*_beat_ at a value that is centered on a bandpass filter with a width of about 12 MHz. This filter provides about 6 dB better S/N than would be obtained by a filter of 50 MHz width (the maximum width that would assure separation of the signal at *f*_beat_ from the signal at *f*_rep_ – *f*_beat_). This provides satisfactory S/N for any of our current measurement needs. A yet narrower filter would provide better S/N but might not be broad enough to accept the signal of a modulated laser; iodine stabilized lasers at 633 nm have a modulation width of 6 MHz, and one commercial 1.52 μm laser has a modulation width of 10 MHz.

If it is necessary to further reduce noise, this can be done by removing the modulation from the beat signal, allowing use of a narrower filter. A suitably fast beat frequency servo should narrow the modulation width, but the effect is not profound for our system. Better results are obtained by adding an AC signal into the feedback system so as to modulate the comb frequency in a manner that cancels the modulation of the test laser. Cancellation is possible by adjusting the amplitude and phase of a sinusoidal voltage output of a phase locked loop. The PLL must be locked to a reference signal which has a fixed phase relationship to the laser modulation. Some modulated lasers provide a reference output that can be used for this purpose. If no such output exists, it is also possible to derive a reference from the beat frequency servo error signal (but performance will not be as good).

We can narrow the linewidth of the beat signal from 6 MHz to about 400 kHz using a PLL. If future measurements require improved S/N, this narrowing of the modulation width will allow us to replace our 12 MHz bandpass filter with a filter of ≤1 MHz width— sufficiently large to comfortably accept the beat signal while providing an additional 11 dB improvement in S/N.

### 2.7 Determining the Mode Order

The mode order *N* must be determined. One way to do this would be to employ a wavemeter, but a suitably accurate wavemeter is expensive, requires periodic calibration, and would require more than one set of optics to cover the visible and IR wavelengths of interest to us. Alternatives based on changing the comb repetition rate [17, 18] are more attractive as a method of determining order, as they avoid the expense and maintenance associated with a separate instrument. Our method is most similar to that of Ref. [17] and is closely analogous to multicolor interferometry [19]. Implementation of these methods is very straightforward when measuring very stable sources. It is somewhat more difficult to implement when measuring the wavelength of typical commercial stabilized lasers, due to the frequency fluctuations of these lasers.

Usually, the frequency of the laser under test is known approximately before beginning the measurement. In fact, the mode order for commercial stabilized lasers is almost always known, since the tolerance on the output frequency is much less than the comb spacing. However, certain assembly errors occasionally cause a commercial laser to have a frequency that is not at the expected order. Even if the exact order is not known, only a narrow range of orders will be realistically possible for a gas laser. For example, the frequency of a 633 nm He-Ne laser will always lay within a known range of 2.8 GHz [20], corresponding to 28 possible values for *N*. Within this range, the order can easily be determined by changing the repetition rate by some amount Δ*f*_rep_. For our system, Δ*f*_rep_ is normally set at 208.22 kHz, as explained previously. In all the following analysis we assume that Δ*f*_rep_<< *f*_rep_.

To determine the mode order, we first use Eq. (2) to find *N*_0_, the value of *N* that gives a result *f*_test_ as close as possible to the expected frequency of the test laser when the repetition rate is *f*_rep_. We next find the order $N_{0}^{\prime }$ which, for repetition rate *f*_rep_ + Δ*f*_rep_, gives a value for *f*_test_ as close as possible to the value obtained with *f*_rep_. If the initial guess *N*_0_ is correct, then the two values obtained for *f*_test_ must be the same (within experimental uncertainty). If the first estimate *N*_0_ is in error by Δ*N*, and if Δ*N* is not too large [more precisely, |Δ*N*| < *f*_rep_/(2Δ*f*_rep_) = 240 orders], then $N_{0}^{\prime }$will be in error by the same amount Δ*N*. Thus one result for *f*_test_ will be in error by Δ*N f*_rep_ and the second will be in error by Δ*N* (*f*_rep_ + Δ*f*_rep_), so the two results of Eq. (2) will disagree with each other by

(3) $$\delta \;f_{\text{test}}=\Delta N\times \Delta f_{\text{rep}}.$$

Therefore the true order must be

(4) $$N=N_{0}-\mathrm{Round}[\delta \;f_{\text{test}}/\Delta f_{\text{rep}}]$$

where “Round” represents rounding to the nearest integer. If there is an error in measuring *δf*_test_ such that the error in *δf*_test_/Δ*f*_rep_ exceeds 0.5, then *N* will be in error by 1. This is never a problem when measuring a laser stabilized by saturated absorption, but it can become a significant problem when measuring certain commercial stabilized lasers, which have much greater frequency fluctuation and drift. Unless the variations of *f*_test_ are somehow measured and corrected, it will be necessary to assume that *f*_test_ is constant, and the variations of *f*_test_ between measurements will appear as errors in *δf*_test_. To avoid an error in determining *N*, it is necessary to tune the comb repetition rate by amounts that are at least twice as large as these frequency fluctuations. Zhang et al. [17] recommend that the repetition frequency be shifted by more than ten times the frequency fluctuations in the laser under test. Our shift of 208 kHz is not as large as recommended, but we can compensate for the relatively small shift as described below.

For our system, we will have an error in *N* unless frequency fluctuations and drift can be kept below Δ*f*_rep_/2 = 108 kHz. This condition can usually be fulfilled but it is not guaranteed. Frequency fluctuations can be reduced by using long sampling times, an efficient solution to the problem if the fluctuations are characterized by a white-noise spectrum. Simply increasing the averaging time will not completely solve the problem for some commercial lasers, which may be subject to frequency variations that are quasi-linear or quasi-periodic on long time scales (ranging from a few minutes up to more than an hour). A more efficient method of averaging out long-term drift is to switch back and fourth several times between two values of *f*_rep_ on a time scale short relative to the time scale of the drifts. For most commercial lasers, reasonable sampling times probably lie between 20 s and 2 min.

As mentioned previously, we shift the repetition frequency by sliding a piece of glass into or out of the free space region of the comb resonator. When performing this test, we servo-control the beat frequency so as to keep *f*_beat_ constant (rather than directly stabilizing the repetition frequency). Mode locking will usually self-initiate when the glass is inserted. On occasion operator intervention is required, but, on average, the process of switching the repetition rate requires less than 20 s.

**Fig. 3:**
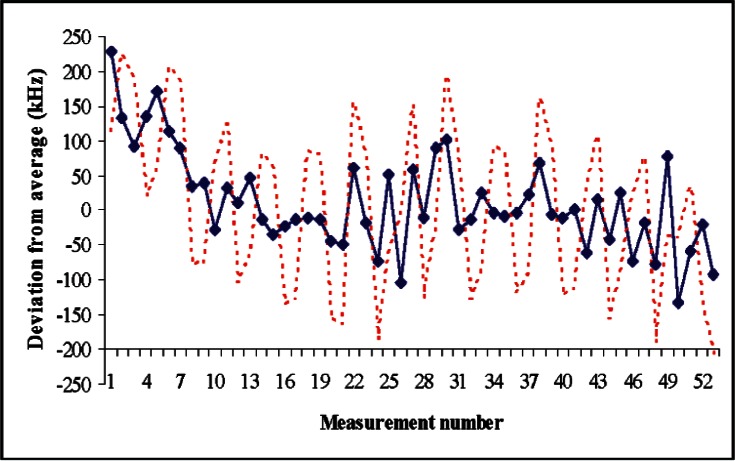
Repeated measurement results for *f*_test_ (deviations from the average value) while switching between two values of the repetition frequency. The solid line shows the correct answer. The dashed line shows what results would look like if the order were misidentified by 1. Uncertainties of the individual frequency measurements are too small to be visible on the graph.

Figure 3 shows repeated measurements of *f*_test_ for a typical commercial laser (633 nm). If *f*_in_ is a frequency measurement with the glass inserted and *f*_out_ is a measurement without the glass, then the data is taken repeating a pattern *f*_in_ *f*_out_ *f*_out_ *f*_in_. Each measurement shown in Fig. 3 is actually a 10 s sample, with two measurements are taken in succession (except for the very first and last point). The sequence *f*_in_ *f*_out_ *f*_out_ *f*_in_ yields two measurements of *δf*_test_ whose average is insensitive to linear drift.

A long-term drift is apparent in the frequency of the test laser. Over the period of the first 10 measurements the average drift between successive readings is about 25 kHz, which will have a non-negligible effect on the results (but is still smaller than typical random fluctuations from one reading to the next, suggesting that a longer averaging time would be beneficial for testing this laser). In Fig. 3, the data points connected by a solid line show the results when the correct order is used in the analysis. The dashed line shows what the results would look like if the order were misidentified by 1. For the dashed line, the frequency clearly changes as we switch between glass-in and glass-out; the clear systematic difference between the readings *f*_in_ and the readings *f*_out_ indicate that the order has been misidentified.

A typical measurement sequence could consist of three repetitions of the pattern *f*_in_ *f*_out_ *f*_out_ *f*_in_—a total of 12 frequency measurements (6 measurements of *δf*_test_). The data of Fig. 3 represents 4 such measurement sequences. The results are unambiguous in the sense that, for each of these 4 measurement sequences, the average value for *δf*_test_ is consistent with 0 and is inconsistent with ± 208 kHz (a change of *N* by ± 1) at a 99.7 % confidence level or better, giving us good assurance that the order has been identified correctly. Thus we conclude that, for this particular laser, a set of three repetitions of the *f*_in_ *f*_out_ *f*_out_ *f*_in_ pattern, with 10 s sample time for each data point, suffices to provide a reliable value for the order *N* that can be obtained in a reasonable amount of time (about 5 minutes).

If the average value of *δf*_test_ is *m* and if the standard deviation of the mean of all measurements is σ, then either neighboring order is excluded with 99 % confidence if

(5) 208kHz−|m|>kσ

where *k* is given by the Student’s t-distribution (*k* = 3.36 for 6 samples and a one-sided confidence interval of 99 %). This provides strong evidence that the order is identified correctly. Equation (5) will always provide a conservative criterion for claiming that the order has been identified correctly, although it may be unnecessarily conservative in some situations where slightly different analyses may be more appropriate.

If all of the measurements shown in Fig. 3 are taken together, the average value of *δf*_test_ is 17 kHz with an uncertainty of 13 kHz, where these values are quite small relative to the fluctuations of the test laser. The good agreement between glass-in and glass-out frequency values provides real-time assurance that the measurements are being done correctly, at a level significantly smaller than uncertainties in the test laser associated with its drift. This is one of several tests we can do to assure that measurements are being done correctly. Of course, this good level of agreement will not verify everything in our measurement process, but it would catch subtle errors in the measurement of *f*_rep_ or gross errors in measurement of *f*_beat_.

In most cases, the procedure above is all that would be needed to distinguish the order. One exception would be if there was very little prior knowledge as to the order, such as when measuring a tunable diode laser. If the true mode order does not lie within the range *N*_0_ ± *f*_rep_/(2Δ*f*_rep_), then the analysis of Eq. (4) breaks down, and ambiguities in possible solutions can arise. (An analogous problem occurs in two-color interferometry when prior knowledge of the measured length is not sufficiently accurate.) If *f*_rep_/Δ*f*_rep_ were exactly 480, so that 481 orders of *f*_rep_ exactly matched 480 orders of (*f*_rep_+Δ*f*_rep_), then the same beat frequency would be predicted for possible solutions differing by 480 orders, and it would be impossible to distinguish the ambiguity. When *f*_rep_ is not exactly an integral multiple of Δ*f*_rep_, the predicted beat frequencies do not repeat exactly at intervals of *f*_rep_/Δ*f*_rep_ orders, and in principle the unambiguous region can be extended. In fact, the sum total of all the data shown in Fig. 3 is sufficiently accurate to relax the requirements on prior knowledge of the wavelength. However, a much smaller set of measurements could efficiently distinguish the order unambiguously, if a series of small frequency shifts is used to perturb the 208 kHz shifts. Because the non-ambiguity interval is given by (*f*_rep_/Δ*f*_rep_), it is clear that we could use a very small shift Δ*f*_rep_ to expand the non-ambiguity interval. A geometric sequence of everdecreasing shifts can be used to expand the non-ambiguity interval to cover the entire visible spectrum or beyond. In practice, these small frequency shifts do not need to be done independently of the large shifts (208 kHz via inserting the glass) that are used to distinguish *N* from *N* + 1; if the 208 kHz shifts are perturbed by additional smaller shifts made in some other manner, it is possible to completely eliminate ambiguity without increasing the total number of required measurements.

## 3. Traceability and Evaluating Measurement Uncertainty

An important aspect of a GPS-based comb system is that it is based on a traceable frequency standard, provided by the GPS system. The traceability of GPS and of GPS-based combs has been discussed in various articles [3, 13–15]. In this section we discuss traceability from the standpoint of the VIM definition [21], “…the property of the result of a measurement or the value of a standard whereby it can be related to stated references, usually national or international standards, through an unbroken chain of comparisons, all having stated uncertainties.” For laser calibrations via a GPS-comb, the unbroken chain of comparisons to national standards can be remarkably short, and the uncertainty budget can be remarkably simple. We will not discuss legal or documentary aspects of traceability.

In the simplest possible measurement system, the frequency of the test laser is determined from Eq. (2):

(2) $$f_{\text{test}}=f_{\mathrm{CEO}}=N\;f_{\text{rep}}\pm f_{\text{beat}}.$$

This equation is valid if no additional frequency offsets are introduced by devices such as acousto-optic modulators (AOMs). In essence, determining *f*_test_ thus depends on determining three frequencies, and the argument for traceability of the measurement rests on two points:

- All frequency determinations are traceable to NIST and to the SI second via a unbroken chain of measurements, with the GPS satellite system playing the central role: (1) a frequency is either measured by a counter, with its timebase provided by the GPSDO, or it is effectively determined by servolocking the frequency to a reference signal derived from the GPSDO (2) the frequency reference for the GPSDO is provided by the signal from GPS satellites and (3) this GPS signal is linked to NIST primary standards (“calibration”) by measurements that are recorded in the NIST GPS Archives [12].
- Our uncertainty budget for laser calibrations using the GPS-comb is entirely dominated by effects that can be quantified by measuring shortterm fluctuations of *f*_beat_ when the comb is compared to a stable laser.

For our application, we need not be concerned with small uncertainties such as the uncertainty of the NIST primary cesium standard; the only uncertainties of any significance for us are those exceeding a few parts in 10^13^. The argument we wish to make is that all of these sources of uncertainty in the GPS-comb system are errors that vary on short time scales, typically less than one or two days. Therefore, the combined magnitude of these effects can be quantified by measuring the apparent variations in a stable test laser for a period of time somewhat longer than 1 day. Because the test laser is not perfectly stable, this test will overestimate measurement errors, but the resulting upper bound on measurement uncertainty is nevertheless sufficient for most needs.

Estimating measurement uncertainty via studying the repeatability of the measurement is a well-established technique often used in dimensional metrology at NIST. However, it often requires years of data to truly sample all sources of error in a typical dimensional measurement system. For the GPS-comb system, we will argue that the required time is much shorter, somewhere between 1 day and 1 week. There are several natural timescales of the GPS system that come into play. One natural time scale is the sidereal day, a timescale that includes effects due to diurnal variations of the ionosphere and diurnal temperature variations. GPS satellites pass overhead twice per day, so that effects associated with individual satellites or with multipath reflections into the antenna will repeat with this period. Another important scale is set by time constants in algorithms that steer the GPSDO local oscillator. Usually these time constants are no more than a few hours, but some exceptional GPSDO systems might update steering only infrequently [15]. It is desirable that the GPSDO manufacturer provide information as to what is the longest timescale associated with their steering algorithms. The natural timescale of the system is either 1 day or, if the steering algorithms employ longer timescales, then it is the longest timescale associated with the steering algorithms. In this article, we will define “short-term” or “long-term” in reference to the longest timescale thus defined. A testing period that samples all errors must be longer than the longest timescale associated with the GPS system and must be longer than 1 day; probably two days of testing would be minimally required to assure that all errors are sampled. We believe that *all* plausible GPSDO errors are short-term and that there are *no* long-term systematic offsets at levels of interest to us (above 1 part in 10^13^). Although it is true that some GPSDO units have much better performance than others in terms of Allan deviation for a particular sampling interval, and many commercial units are simply not well designed for use as a frequency standard, the authors are aware of no instances where commercial units give systematic long-term frequency offsets. Consequently a study of short-term repeatability will suffice to quantify all uncertainties associated with the GPSDO.

The argument presented above regarding time scales is not *strictly* true; some relevant time scales are much longer. For example, the 11-year sunspot cycle could also have some bearing on the measurements; increased solar activity may reduce short-term stability of the GPS signal and will thus increase measurement uncertainty. Any such degradation of the GPS signal will be seen as increased Allan deviations as recorded in the NIST archives. If the Allan deviation significantly increases at some point in time, it will be necessary to increase estimated measurement uncertainty and may become necessary to re-quantify the short-term stability of the system by comparison to a stable laser. Significant changes in the environment of the antenna—including seasonal changes in nearby foliage—might also slightly degrade performance on a long time scale, requiring re-quantification of the short-term stability.

With the basic link to the SI unit provided by GPS, the primary traceability issue is in evaluating uncertainty due to short-term fluctuations. For measurements at the 10^−12^ level, it is the fluctuations in *f*_rep_ that are of primary importance. These fluctuations arise primarily from fluctuations in the received GPS signal (including effects such as fluctuations in the clocks of the GPS satellites, varying atmospheric delays, and multipath reflections), as modified by the smoothing provided by the GPSDO. If *f*_rep_ is phase locked to a reference frequency derived from the GPSDO (our normal mode of measurement), then there are additional fluctuations in *f*_rep_ due to imperfect performance of the servo. If *f*_beat_ rather than *f*_rep_ is stabilized, then *f*_rep_ must be measured, and there are additional fluctuations due to the timing resolution of the frequency counter. Regardless of which scheme is employed, it is possible to put an upper limit on the uncertainty associated with short-term stability. Ideally, this would be done by comparing the GPSDO/comb system to perfect local oscillator—an ultra-stable laser. But useful information regarding GPSDO/comb stability can often be obtained by comparing it to a less-than perfect laser, such as an iodine stabilized laser or possibly even a polarization stabilized laser (although a polarization stabilized laser is not sufficiently stable for implementation of some diagnostics to be described later). When comparing the comb to an imperfect laser, the measurements quantify the combined instabilities of the two systems and thus provide an upper bound on the stability of the GPS-comb. There is no inherent reason that any laser cannot be used for the measurement, as long as the upper bound is low enough to serve the needs of the calibration lab. The laser employed for the stability measurement need not be calibrated prior to the measurement. In principle, if a laboratory does not own an iodine stabilized laser but iodine stabilized lasers are sent to the lab for calibration, then a customer laser could be used to carry out the stability measurements. (Of course, if a customer needs precise knowledge of the short-term Allan deviation of his/her iodine stabilized laser, this information cannot be obtained without better quantification of the GPS-comb short-term stability.)

We have measured comb stability by comparing it to one of our iodine stabilized lasers. To achieve the best possible results, the laser sits undisturbed in a temperature-controlled laboratory and is warmed up for many hours prior to beginning the measurement. Also, the GPSDO should be fully warmed up and finished with any self-survey that may be performed to determine the location of the antenna. Even under these circumstances, drift of the test laser frequency may not be insignificant; if the drift is incorrectly attributed to the comb, it can make the comb appear to be less stable than it actually is. Nevertheless, the amount of drift is small enough to satisfy our needs.

**Fig. 4:**
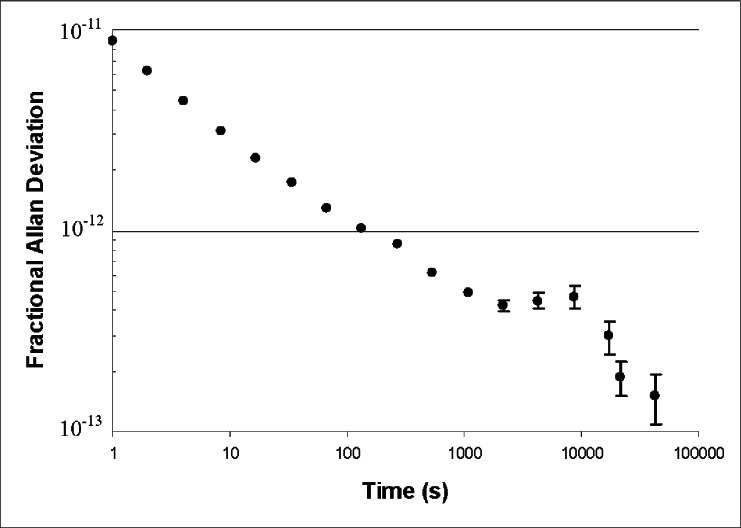
Allan deviation of *f*_beat_, comparing the comb to an iodine stabilized laser. Error bars corresponding to the standard uncertainty are shown for longer averaging times, where the error is large enough to be significant.

Figure 4 shows the Allan deviation of *f*beat measured over a period of 6 days. The measurement quantifies the combined effect of short-term fluctuations in the GPSDO, the comb, the iodine stabilized laser, and all ancillary equipment such as frequency counters. The shape of the curve is very similar to the manufacturer’s published results for this GPSDO, although the magnitude of the observed Allan deviation is smaller than shown by the manufacturer for sample times longer than 10 s. The results of Fig. 4 provide a useful upper bound on fluctuations of the comb system, demonstrating that we can achieve good accuracy for measuring times in excess of 100 s, where the Allan deviation falls below 1 part in 10^12^.

During the period of time when these data were collected (July 27 to August 1, 2006), there was little sunspot activity and the NIST archives do not indicate any unusual errors in the GPS signal. The results of Fig. 4 are indicative of what can be expected under similar near-ideal conditions, but measurement uncertainty should be re-evaluated when the NIST archives show decreased stability of the GPS signal.

**Fig. 5:**
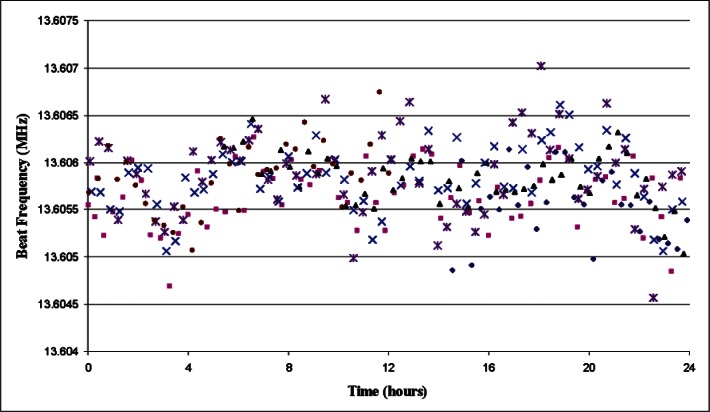
Beat frequency vs. time of day (hours past midnight). Measurements were taken over a period of six days. The different symbols in the graph correspond to data from different days. Uncertainties of the individual beat frequency measurements are too small to be visible on the graph.

The data shows that for measurement times longer than approximately 1000 s the Allan deviation falls below 5 × 10^−13^ and remains below this value up to the longest time measured. If we can argue that the long-term average of these measurements has an uncertainty much less than 5 parts in 10^13^, then we can conclude that the standard uncertainty of measurements in excess of 1000 s duration is on the order of 5 × 10^−13^. More direct evidence of stability is given in Fig. 5, which shows results for beat frequency measurements between the comb and laser with 22 min (1340 s) averaging time. The data is graphed as a function of time of day, and was collected over a period of six days. The data suggests that there may be small correlations between the measured frequency and time of day, but any such systematic fluctuations are unlikely to exceed 1 kHz peak-to-valley.

Because of these systematic variations on a time scale of many hours, the 22 min Allan deviation may slightly underestimate the true uncertainty of a 22 min measurement. The long-term variations are reflected in the fact that the Allan deviation does not decrease as the averaging time is increased from about 1000 s to 10 000 s (but decreases again for sample times in excess of six hours, dropping to 1.5 × 10^−13^ at 12 hours).

The sample standard deviation of the data in Fig. 5 is 370 Hz (7.8 × 10^−13^), and 95 % of the points are within 730 Hz of the mean. This suggests that a reasonable *k* = 1 estimate of the uncertainty for a measurement averaged over 22 min is 370 Hz (7.8 × 10^−13^), where this value might be slightly inflated by (possible) drift of the iodine stabilized laser. To this could be added the expected uncertainty (relative to the SI unit of time) of the six-day average, but in the absence of serious satellite failure, six-day averages are almost always in error by less than 1 part in 10^13^ and thus have no effect on the result. Indeed, the NIST archives show that the six-day average during this test was in error by only 1 × 10^−14^, and the additional error added by our GPSDO over a period of 6 days should be well less than the 1.5 × 10^−13^ Allan deviation observed at the longest sampling times. Even with the most pessimistic possible assumptions, these additional uncertainties would have almost no effect on the overall uncertainty for 22 min averages, increasing the uncertainty from 7.8 × 10^−13^ to 7.9 × 10^−13^.

In a similar manner, any laboratory with access to an iodine stabilized laser can estimate short-term uncertainties of their system, without appeal to an outside agency such as an NMI. Once again, we reiterate that these short-term uncertainties capture all the important sources of uncertainty in the measurement process and that no additional measurements are needed to evaluate the uncertainty of the system. Thus, documentation of the short-term performance, combined with the traceability of the GPS signal, might form a basis for claiming traceable comb-based measurements with a rigorously evaluated uncertainty.

The 370 Hz standard deviation of the data in Fig. 5 should be a true measure of the uncertainty for 22 min sampling. Essentially the same sample standard deviation is obtained for 22 min averages whether data is averaged from a single day or for the full six day period. If the comb could be compared to a perfect stabilized laser, and if sunspot activity were constant, there would be every reason to believe that a similar standard deviation would be calculated from data taken over a period of six years as was obtained for just six days. Our estimated expanded uncertainty (1.8 × 10^−12^ with *k* = 2) should be reliable as long as the NIST archives don’t indicate any unusual problems.

Finally, note that there is no uncertainty component assigned to the measurement result (*f*_test_) arising from uncertainty in the determination of *N* in Eq. (2). If the procedure for determining *N* as given in Sec. 2.7 were applied in precisely the manner that was described, then about 1 measurement in 100 would be in error by an amount exceeding 10^5^ *u*, where *u* is the standard uncertainty we have assigned to the measurement. (In reality, a misidentified order would normally be an unexpected result that would undoubtedly prompt additional measurements for verification.) The same situation arises in multicolor interferometry, and it has long been recognized that this has no bearing on an uncertainty budget. Misidentification of *N* is classified as a “blunder,” outside the uncertainty budget. There are many other blunders that can also occur in a comb measurement, where the term “blunder,” within the context of metrology, refers to an avoidable mistake but not necessarily a large mistake [22]. For example, the GPS signal might be lost, or the beat frequency can be miscounted if the S/N is low. These blunders will often give rise to small errors but, as in the case of misidentifying *N*, can potentially cause errors many orders of magnitude larger than the standard uncertainty. Regardless of the size of the error, they have no bearing on an uncertainty budget, and they do not relate to traceability as defined by the VIM. Blunders are discussed in the next section.

## 4. Blunders

Blunders are not explicitly discussed in the VIM definition of traceability. Nevertheless, for the GPS-comb system, blunders represent the primary impediment to achieving confidence in measurement results at our claimed uncertainty, where “blunders” include both misuse of equipment (blunders by an operator) and blunders by the designers of equipment. (Beyond simply being “out of spec,” poorly designed equipment might not even function in accord with its intended operating principles.) Therefore it is desirable to verify, as far as possible, that blunders have not occurred.

### 4.1 Blunders in the Design or Use of the GPSDO

If a laboratory were to use its own cesium clock as a standard, not tied to GPS, it might be necessary to send it to an NMI for calibration, but if we use the clocks of the GPS satellites, calibrations are performed automatically. Thus, the system does not need to be calibrated to avoid long-term offsets. Short-term performance of the GPSDO can be verified as described already. Thus, it is probably not necessary to send a unit to an NMI for calibration. Furthermore, although there can be several benefits from obtaining an NMI calibration, it may still be necessary to locally verify short-term performance of the entire GPS-comb system, including effects such as antenna placement which can only be evaluated by *in situ* testing.

As stated previously, we are not aware of a GPS unit that has ever been produced that has a long-term frequency offset, if “long-term” is defined as described previously. (By contrast, a systematic *time* offset can easily occur in a GPS system.) There is, nevertheless, a practical problem in defining “long-term,” because this can only be done if the manufacturer provides reliable information regarding operation of their unit. To some extent, the manufacturer’s information can be supplemented by looking at results such as shown in Fig. 4, where the structure of the plateau between 1000 s and 10 000 s suggests that “long-term” must be longer than 10 000 s. Certainly if a similar plateau or an increase in the Allan deviation occurred at the longest sampling times, this might indicate that the test of short-term stability is not long enough to capture all sources of error. Of course, the possibility that the software makes some steering corrections once every two weeks or even once per year can not be ruled out by data obtained over a six-day period; it is still of interest to verify from the manufacturer that such infrequent steering corrections do not occur.

The plateau in Fig. 4 also conveys another important piece of information. The shape of the curve is characteristic of the steering algorithm for this particular model GPSDO (that is, the shape of the curve corresponds to published data for this model) and bears no resemblance to what would occur if the rubidium clock were not controlled by GPS. The characteristic shape seen in the diagram proves that the critical GPSDO subsystems are performing correctly. The shape would not be seen if GPS steering were disabled by an operator blunder or by a broken line to the antenna. It would not be seen if the GPS steering servo or the rubidium cell had failed. One would expect that a GPSDO will include diagnostics that warn a user of such catastrophic failures, but even in the absence of these diagnostics the shape of the curve proves that major failures (that could give rise to long-term offsets) have not occurred.

It is difficult to imagine other plausible scenarios that would give a long-term error that is small enough to go undiscovered but large enough to affect our measurements at the 10^−12^ level. Nevertheless, in principle a design blunder or firmware bug might produce such a small long-term offset. The best defense against such errors is for the manufacturer to carefully compare each new model (or new firmware update) to a model whose operation has been previously verified. The new model might also be sent to a NMI for testing, and this has a good chance of uncovering design blunders. In-house comparisons by the manufacturer have a slight advantage because they can be carried out over longer periods of time, but in some manner the manufacturer should also directly or indirectly compare his model to national standards. If these modest and reasonable measures are carried out to assure the integrity of a new model, it is difficult to see how a hidden systematic offset would ever occur, even at the 10^−14^ level. It is probably more logical for a manufacturer to thus verify operation of his product line than for every individual unit to be sent for calibration to an NMI.

An alternate method of testing for subtle GPSDO errors might be to compare to another well-characterized frequency standard. One possibility is to compare to a second GPSDO from a different manufacturer, using an independent antenna, and show that both units give the same frequency. We have tried this approach with limited success for two units that share the same antenna (thus not a perfect test). The two units are very different in operation—one uses a rubidium local oscillator and the second, from a different manufacturer, used quartz—and consequently we can argue that there should be very little correlation in the errors of the two units (other than errors associated with the common antenna). Unfortunately, the quartz unit does not have sufficiently good stability to provide a useful upper bound on short-term performance, but for averaging times approaching 1 day, where the fractional difference in frequency of the two units falls below 3 × 10^−13^, this test provides further confidence that our GPSDO is working properly. However, the test is neither as comprehensive nor as sensitive as is the comparison to an iodine stabilized laser.

### 4.2 Blunders in Determining Comb Frequencies *f*_rep_ and *f_CEO_*

The short-term stability of the comb is evaluated along with the stability of the GPSDO as described previously. A remaining question is whether the comb could be subject to systematic offsets even if the GPSDO is operating correctly. For example, there could be errors in the locking of *f*_rep_ and *f_CEO_* or in the measurement of these frequencies.

One of the more likely sources of trouble would be poor S/N levels in the *f* – 2*f* interferometer, leading to an incorrectly controlled value for *f_CEO_*. The accuracy of *f_CEO_* can be checked by using an independent frequency counter to measure *f_CEO_*, although there is some danger that noise might generate the same error in the counter as in the electronics controlling *f_CEO_*. This possibility can be eliminated if the counter measurements are unchanged when the signal is filtered with filters of differing bandwidths. Confidence in proper operation will also be increased if it is observed that the measured value for *f*_test_ does not change when the *f* – 2*f* interferometer is slightly misaligned, thus reducing the S/N. Finally, a good test of overall system performance, particularly sensitive to errors in *f_CEO_*, is to demonstrate that the measured value of *f*_test_ remains unchanged if the sign of *f_CEO_* is reversed by changing the polarity of the servo. By switching the polarity several times, it is possible to distinguish a systematic offset from instability of the test laser. This is a worthwhile test because consistent results will not be obtained unless both *f_CEO_* and *f*_beat_ are being measured correctly and the signs of the two frequencies are understood correctly.

It should also be verified that the repetition frequency *f*_rep_ is being controlled correctly (or, if *f*_beat_ is controlled, that *f*_rep_ is measured correctly.) Actual miscounting of *f*_rep_ is highly unlikely, because S/N is very large. Furthermore, a miscount of 1 in a sampling time under 10 s would be a large error that would be immediately obvious. One can imagine slow thermal-dependent phase shifts that would cause more subtle errors—hence more difficult to detect—but even these errors should be detected by the multi-day stability measurements described previously. Nevertheless, it is worthwhile to verify proper operation by measuring *f*_rep_ directly using an independent frequency counter, using the GPSDO as an external timebase for the counter. (An error in *f*_rep_ due to an error in the GPSDO frequency would not be revealed by this test, but verification of GPSDO performance has already been discussed.) As mentioned previously, the frequency counter used for this test must have good timing resolution and accuracy, and long measurement intervals will be required if the measurement is to achieve a relative uncertainty of 10^−12^ (which is an uncertainty of 10^−4^ Hz in *f*_rep_). For example, a 100 ps timing resolution with a sample time of 100 s is only nominally sufficient to achieve the desired accuracy. Averaging shorter samples may not achieve comparable accuracy if there are systematic timing offsets in the counter. For suitably long samples, verifying that the average result is independent of sampling time can be used to argue that any such offsets are not affecting results.

Another useful operational test is to verify that the same value for *f*_test_ is obtained if *f*_rep_ is servolocked to a reference or if *f*_beat_ is servolocked while *f*_rep_ is measured. (Similarly, if *f*_rep_ is controlled but a synthesizer is used to generate different repetition frequencies, consistency of measurement results obtained with different repetition frequencies will provide good evidence that no blunders are causing bad results.) Self-consistency of the measurements of *f*_test_ also provides evidence that the beat frequency is being measured correctly. The beat frequency measurement can also be checked more directly, as described below.

### 4.3 Blunders in Beat Frequency Measurement

There is some danger that poor S/N ratio, electrical interference, amplifier oscillation, or similar effects can degrade the measurement of *f*_beat_. A simple method sometimes used to verify proper counting is to misalign the comb and laser beams so as to decrease the S/N ratio by more than 5 dB; if this does not change the measured beat frequency then it is apparent that there are no difficulties in the measurement due to poor S/N. (Possible electrical interference problems must be checked separately.) As an alternative to this approach, we have implemented a real-time method for verifying proper frequency counting. We simultaneously measure two beat signals, one at some frequency *f* and the second, arising from interference of the next comb order, at frequency *f*_rep_ – *f*. When the sum of the two signals is exactly *f*_rep_, this provides an excellent real-time indicator that there are no problems with the beat frequency measurement.

If the two frequencies are sampled for 1 s and the two counters disagree by 1 Hz, this is indicative of 1 miscounted cycle. We use external arming of the counters to guarantee simultaneous measurements of the beat signals, and under these conditions the counters should add exactly to *f*_rep_. (Note that advanced counters may employ noise-reduction techniques [23, 24] that, if not disabled, can complicate simultaneous measurements—as is particularly evident when measuring a frequency modulated laser.) Actually, a few miscounts could be tolerated, as this would not affect results at the 10^−12^ level, but one should be aware that the actual number of miscounts might be significantly larger than indicated by the failure of the summation to match *f*_rep_, because errors in the summation due to miscounting associated with white noise tend to cancel. (The errors never cancel completely, but they will cancel on average if the bandpass widths of the two filters are identical and the center frequencies of the two filters add to *f*_rep_.)

### 4.4 Summary—How to Guarantee That Measurement Blunders Do Not Occur

In summary, we believe that a laboratory can avoid almost any imaginable measurement blunder if certain steps are taken.

- The NIST GPS archives should be consulted to assure that the GPS system is operating within normally expected uncertainty levels.
- It must be verified that the GPSDO faithfully reproduces the GPS signal without long-term offsets. As discussed previously, this can be assured if (1) the manufacturer has directly or indirectly compared the specific model used for the comb measurements (and the specific firmware) to national standards and (2) the operator monitors GPSDO diagnostics to assure that there is not a system failure (such as failure to lock to the GPS signal due to RF interference [15]). Also, it will build confidence if the Allan deviation, such as shown in Fig. 4, is consistent with expectations (based on manufacturer’s specifications and data) for the particular model in use.
- After verifying that the GPS signal and the GPSDO are operating correctly as described above, then the primary remaining concerns are that *f*_rep_, *f*_beat_, and *f_CEO_* might not be controlled and measured correctly. The timebase of these measurements is supplied by the GPSDO and thus has been verified, and a number of strategies have been discussed that will uncover errors in the determination of *f*_rep_, *f*_beat_, and *f_CEO_* due to miscounting or faulty servo performance.
- Probably the best way to demonstrate that no blunder has been made in a particular calibration is to look for internal consistency between various ways of performing the measurement, including measurements with the offset frequency polarity reversed and with different repetition frequencies. Good consistency demonstrates that the order number is known, shows that there are no significant errors in measuring *f*_beat_ or *f_CEO_*, and provides additional evidence that imperfect frequency synthesis does not introduce errors in *f*_rep_ that vary nonlinearly with frequency. It also verifies that the operator is not making calculation errors.
- An additional source of error can be very important, even when the tests above demonstrate proper operation of the comb/GPSDO and demonstrate that the frequency counting is error-free. Even when the current frequency of the test laser is being measured absolutely correctly, it must be further verified that the *current* frequency has not been perturbed away from the *normal* operating frequency. The frequency of the test laser can potentially be shifted due to beam reflections re-entering the laser (optical feedback). Reflections will reduce the apparent stability of the test laser and might shift the average value of *f*_test_. Reflections are particularly likely from the surface of the photodetector; this surface should always be tilted so that the lens focusing the laser beams onto the detector does not form a cat’s eye reflector. A Faraday isolator or an AOM can be used to greatly reduce reflections.

Optical feedback is, in fact, a common source of problems when calibrating a laser against the comb. If there is any concern that feedback might be affecting results, this can be tested by inserting a 0.3 optical density filter into the beam from the test laser. The filter will substantially change the feedback, attenuating any possible reflected amplitude by a factor of two (power by a factor of 4). The filter will also shift the phase of the reflected light. If the filter reduces the size of frequency fluctuations or causes a statistically significant shift in the average frequency, then feedback is clearly a problem. Otherwise, we can conclude that feedback is not causing significant errors. The filter must be tilted slightly so that a specular reflection from the surface does not cause feedback.

A final note: if a laser has a two-frequency output (such as Zeeman-stabilized lasers intended for use in displacement interferometers) there is a danger that the measured frequency is not what is needed for the intended application. The customer must specify which frequency component is relevant to his/her needs. (For a heterodyne displacement interferometer, it is the component in the variable-length arm of the interferometer.)

## 5. Concluding Remarks

We have described the comb system used in PED, which is our most accurate realization of the unit of length as embedded in laser vacuum wavelength. The PED comb has never been directly compared to standards from other NMIs. An indirect comparison can be inferred through previous international comparisons of our iodine stabilized lasers, but the uncertainty of this indirect comparison is an order of magnitude above our claimed uncertainty for comb measurements. Furthermore, the central pieces of equipment used in the experiment, the GPSDO and the comb, have never been calibrated. In spite of these facts, we can be fully confident of our uncertainty claim. Although it is never possible to rule out every conceivable error of a measurement system, we can realistically establish a degree of confidence in our comb measurements that is greater than our confidence in many other types of measurements carried out within PED, even including measurements whose uncertainties have been confirmed by direct international intercomparisons. Confidence in comb measurements can be significantly higher than for many other types of measurements because of the great strength of the internal consistency tests, which provide continuous assurance that the measurement is working properly.

In principle, a portable comb referenced to a portable cesium frequency standard can be used to establish international equivalence of comb systems; it can provide high-accuracy, realistic, in situ testing of a complete GPSDO-based comb system, including the effect of GPS antenna performance as mounted in its particular environment. At the present time, however, there are no portable systems in use for direct verification of comb performance. The expense associated with maintaining such systems and carrying out the comparisons is difficult to justify. If it is indeed possible (as claimed here) to develop full confidence in a system via internal consistency testing, while relying on GPS to maintain the link to international standards, then there is little justification for deployment of a portable system to prove international equivalence of comb systems with modest uncertainty claims.

Our confidence in our link to international standards is based on the traceability provided by the NIST GPS archives, and also based partly on the fact that GPSDO units similar to ours have been compared to NIST standards (even though our particular unit has not). Given the validity of this link, we have argued that short-term errors in the system are the only errors of significance, and we can establish an upper limit on our measurement uncertainty via comparison to an iodine stabilized laser. Tests have been described to rule out the possibility of blunders or system failures, leaving few remaining plausible sources of undiscovered problems. Short of exotic errors such as deliberate sabotage, blunders that are not completely implausible might include firmware bugs in the GPSDO or analysis errors. If firmware problems in our system have gone undetected, they must occur on time scales exceeding our six-day test of short-term stability. Almost any measurement system could in principle have firmware errors that would appear only infrequently, making them very difficult to detect by a test (or by an interlaboratory measurement comparison) of finite duration, and we have little choice but to accept this small risk. Likely analysis blunders, such as sign errors or an error typing data into a spreadsheet, will be uncovered if internal consistency is verified in the manner that has been described. More subtle analysis blunders that would not be immediately evident are largely confined to rounding errors, not a major concern for modern computers.

For our measurements, the greatest danger is that a blunder will affect results at the 10^−12^ level but will not be large enough to be apparent from measurements of iodine stabilized lasers, where the answer is known with an uncertainty of a few parts in 10^11^ if the laser is operating properly. Thus, the blunders must fall within a very specific range if the system gives a plausible but incorrect result for the calibration of an iodine stabilized laser. For example, a sign error can easily occur when accounting for the frequency shift of an AOM used as an optical isolator, but the resulting error would be obvious if an iodine stabilized laser were measured. Smaller, subtler errors would be more likely in a more sophisticated system. A system that uses a cesium clock will have more possibilities for errors or blunders than will a GPSDO, even though it is potentially capable of higher accuracy. When the measurement can be embodied with the simplicity of Eq. (2) and all frequencies are tied to the GPSDO, the list of plausible blunders is short, and tests can be devised for any blunder that can be anticipated.

Just as we have confidence in our measurements, we can have good confidence in the results from other laboratories if they follow procedures similar to what has been described here. Arguably, anyone in the world can receive a GPS signal and use it to calibrate lasers over a wide range of wavelengths with good confidence in the results. The basic traceability is delivered by the GPS system, and additional internal testing can verify performance of a particular piece of equipment, quantify short-term uncertainty, and demonstrate competence of personnel. The laboratory can carry out tests to verify operation at a level appropriate to those lasers that they are calibrating—on the order of 10^−12^ for lasers stabilized by saturated absorption or ≤ 10^−9^ for polarization or Zeeman stabilized lasers. There is no clear need for additional testing or interaction with an NMI to prove competence. An interlaboratory comparison of some form is always helpful to instill confidence, but it is not clearly necessary. Also, it may be possible to achieve very good confidence in the measurement using a comparison whose uncertainty is not sufficiently low to fully test the uncertainty of the comb-GPS system [3]. The VIM definition of traceability can be satisfied simply by quantifying and documenting short-term errors while relying on NIST calibrations of the GPS system to provide a link to the SI second.

## Footnotes

1 Not all acronyms will be defined in the text, but definitions can be found in Appendix A. Following reorganization, PED is now the Mechanical Metrology Division.

2 Certain commercial materials and equipment are identified in order to specify adequately experimental procedures. In no case does such identification imply recommendation or endorsement by the National Institute of Standards and Technology, nor does it imply that the items identified are necessarily the best available for the purpose.

## 6. Appendix A: Acronyms

Below are definitions of acronyms used in this paper (including several acronyms that are in such widespread use that they might not need definition but are nevertheless included for completeness).

AOM: acousto-optic modulator
BBO: beta barium borate
CEO: carrier-envelope offset
GPS: global positioning system
GPSDO: GPS disciplined oscillator
IR: infrared
NIST: National Institute of Standards and Technology
NMI: National Metrology Institute
PED: Precision Engineering Division, NIST
PI: proportional-integral
PLL: phase locked loop
PPLN: periodically poled lithium niobate
RBW: Resolution Bandwidth
RF: radio frequency
RMS: Root mean square
SI: Système international d‘unités (the international metric system)
S/N: signal to noise, or signal to noise ratio
VIM: Vocabulaire International de Métrologie (International Vocabulary of Metrology.)
